# Enhanced exosome secretion in Down syndrome brain - a protective mechanism to alleviate neuronal endosomal abnormalities

**DOI:** 10.1186/s40478-017-0466-0

**Published:** 2017-08-29

**Authors:** Sébastien A. Gauthier, Rocío Pérez-González, Ajay Sharma, Fang-Ke Huang, Melissa J. Alldred, Monika Pawlik, Gurjinder Kaur, Stephen D. Ginsberg, Thomas A. Neubert, Efrat Levy

**Affiliations:** 1 Center for Dementia Research, Nathan S. Kline Institute for Psychiatric Research, Orangeburg, NY 10962 USA; 2 Cell Biology and Skirball Institute of Biomolecular Medicine, NYU Langone School of Medicine, New York, NY 10016 USA; 3 Department of Psychiatry, NYU Langone School of Medicine, New York, NY 10016 USA; 4 Department of Neuroscience & Physiology, NYU Langone School of Medicine, New York, NY 10016 USA; 5 Neuroscience Institute, NYU Langone School of Medicine, New York, NY 10016 USA; 6 Department of Biochemistry & Molecular Pharmacology, NYU Langone School of Medicine, New York, NY 10016 USA

**Keywords:** Down syndrome, endosome, extracellular vesicle, exosome, CD63, rab35

## Abstract

A dysfunctional endosomal pathway and abnormally enlarged early endosomes in neurons are an early characteristic of Down syndrome (DS) and Alzheimer’s disease (AD). We have hypothesized that endosomal material can be released by endosomal multivesicular bodies (MVBs) into the extracellular space via exosomes to relieve neurons of accumulated endosomal contents when endosomal pathway function is compromised. Supporting this, we found that exosome secretion is enhanced in the brains of DS patients and a mouse model of the disease, and by DS fibroblasts. Furthermore, increased levels of the tetraspanin CD63, a regulator of exosome biogenesis, were observed in DS brains. Importantly, CD63 knockdown diminished exosome release and worsened endosomal pathology in DS fibroblasts. Taken together, these data suggest that increased CD63 expression enhances exosome release as an endogenous mechanism mitigating endosomal abnormalities in DS. Thus, the upregulation of exosome release represents a potential therapeutic goal for neurodegenerative disorders with endosomal pathology.

**Electronic supplementary material:**

The online version of this article (doi:10.1186/s40478-017-0466-0) contains supplementary material, which is available to authorized users.

## Introduction

A dysfunctional endosomal pathway and abnormally numerous and enlarged early endosomes are found within vulnerable Alzheimer’s disease (AD) neurons early in life [7]. These characteristics are also seen in Down syndrome (DS) [10], a genetic disorder caused by trisomy of human chromosome 21 that leads to early-onset AD [55]. Early endosomal abnormalities correlate with developmental brain abnormalities and intellectual disabilities in DS patients [35, 36, 54]. Dysfunction within the endosomal pathway, which occurs in several neurodegenerative disorders [53] in addition to AD and DS, results in the accumulation of material in neuronal endosomes with subsequent neuronal vulnerability and degeneration [39]. Early endosomal changes have also been observed in vitro in fibroblasts derived from DS individuals [8] and in neurons of well-established mouse models of DS [9, 26, 44]. In this study we have utilized the trisomic mouse model Ts[Rb(12.17^16^)]2Cje [52] (hereafter called Ts2), which presents phenotypic and pathological features [26, 29, 52] similar to the extensively studied Ts65Dn mouse model [9, 15, 20, 22], but has a genetic configuration with advantages in breeding [26, 32, 52].

Early endosomes are the first vesicular compartment along the endocytic pathway, which internalizes cargoes for delivery to late endosomes/multivesicular bodies (MVBs) for either degradation in lysosomes or for release into the extracellular space via exosomes. Exosomes are extracellular vesicles (EVs) formed as intraluminal vesicles (ILVs) by inward invagination of the membrane of MVBs, which are released into the extracellular space upon fusion of MVBs with the plasma membrane [12]. ILVs formation is regulated by the endosomal sorting complexes required for transport (ESCRT) machinery as well as by an ESCRT independent system that includes proteins from the tetraspanin family [11]. While most tetraspanins are present in the plasma membrane, CD63 is uniquely enriched in the membrane of MVBs [42]. Intracellular trafficking of MVBs towards the plasma membrane is regulated by several Rab GTPases and SNARE proteins [17]. Among them, rab35 likely plays a role in the docking of MVBs to the plasma membrane [24].

We investigated whether endosomal abnormalities affect the exosome secretory pathway in DS and explored the molecular mechanism underlying changes in exosome secretion in vivo in the brain of DS patients and the Ts2 murine model, and in human DS fibroblasts grown in vitro. The results support the hypothesis that in neurodegenerative disorders with endosomal-lysosomal dysfunction, such as DS, exosome secretion serves as a disposal mechanism of toxic material that is abnormally accumulated in endosomal compartments. Given that endosomal abnormality causes neuron degeneration [39], mitigation of this pathology by enhanced exosome release can be expected to be protective and a target for therapeutic intervention.

## Materials and methods

### Mice

Ts[Rb(12.1716)]2Cje (Ts2) [52] and normal disomic (2N) littermates were studied at 3, 8, 12 and 24 months of age. Both females and males were used for all analyses. All animal procedures were performed following the National Institutes of Health guidelines with approval from the Institutional Animal Care and Use Committee at the Nathan S. Kline Institute for Psychiatric Research.

### Human brain tissues

Postmortem samples of Brodmann area 9 (BA9) obtained from human DS and control subjects (Table 1) were kindly provided by Dr. Jerzy Wegiel, Director, Brain Bank for Developmental Disabilities and Aging, Institute for Basics Research in Developmental Disabilities, Staten Island, New York. Tissue accession and use protocols were approved by the Nathan S. Kline Institute.

**Table 1:** Frozen samples of Brodmann area 9 from control (2N) and DS subjects

#	Genotype	Age	Gender	PMI (h)
1	DS	43	F	>24
2	2N	31	M	3
3	DS	43	F	NA
4	2N	32	M	14
5	DS	59	M	>24
6	2N	59	M	6
7	DS	59	F	NA
8	2N	67	F	4
9	DS	60	F	22.5
10	2N	68	F	3

Age is expressed in years

*PMI* post mortem interval, *NA* not available

### EVs isolation from brain tissue

EVs were isolated from frozen samples of cortical human brain region BA9 and from the right murine hemibrains (without the cerebellum and the olfactory bulbs). In each experiment, EVs were simultaneously isolated from a brain of either a DS patient or a Ts2 mouse and from an age-matched 2N control. Brain EVs were isolated and purified as we have previously described [40, 41]. Briefly, frozen brain tissues were treated with 20 units/ml papain (Worthington, Lakewood, NJ) in Hibernate A solution (HA, 3.5 ml/sample; BrainBits, Springfield, IL) for 15 min at 37 °C. The brain tissue was gently dissociated in 6.5 ml of cold HA supplemented with protease inhibitors, centrifuged at 300 g for 10 min at 4 °C to discard the cells, and the supernantant was sequentially filtered through a 40 μm mesh filter (BD Biosciences, San Jose, CA) and a 0.2 μm syringe filter (Corning Life Sciences, Teterboro, NJ). The filtrate was sequentially centrifuged at 4 °C, at 2000 g for 10 min and 10,000 g for 30 min to discard membranes and debris, and at 100,000 g for 70 min to pellet the EVs. The EVs pellet was resuspended in 60 ml of cold PBS (Thermo Fisher Scientific), and centrifuged at 100,000 g for 70 min at 4 °C. The washed EVs pellet was resuspended in 2 ml of 0.95 M sucrose solution and inserted inside a sucrose step gradient column (six 2-ml steps starting from 2.0 M sucrose up to 0.25 M). The sucrose step gradient was centrifuged at 200,000 g for 16 h and fractions were collected from the top of the gradient. The fractions were diluted in cold PBS and centrifuged at 100,000 g for 70 min. Sucrose gradient fraction pellets were resuspended in 30 μl of cold PBS.

### Quantification of EVs levels in the brain

Protein levels from the sucrose gradient purified fractions b, c and d, were quantified using the BCA Protein Assay Kit (Thermo Fisher Scientific). Acetylcholine esterase (AChE) activity assay, commonly used to quantify exosomes, was also performed on fractions b, c, and d, as we have previously described [40, 41]. Total EVs protein levels and EVs AChE activity were normalized to total brain sample protein content. AChE activity was additionally normalized to total EVs protein.

### Western-blot analyses

Brain homogenates (10 μg protein), fibroblast lysates (10 μg protein), and EVs proteins (15 μl of the lysate corresponding to 25% of the EVs lysate total volume), were separated by 4–20% tris-glycine gel electrophoresis (Criterion precast gel, Bio-Rad, Hercules, CA) and transferred onto PVDF membranes (Immobilon, EMD Millipore, Billerica, MA). Membranes were incubated with antibodies to CD63 (1:250, Cat# sc-15363, Santa Cruz Biotechnology, Dallas, TX), Alix (1:1000, Cat# ABC40, EMD Millipore), TSG101 (1:1000, Cat# 4A10, GeneTex, Irvine, CA), Flotillin-1 (1:1000, Cat# 610821, BD Biosciences, San Jose, CA), Flotillin-2 (1:1000, Cat# 610384, BD Biosciences), rab35 (1:1000, Cat# 9690, Cell Signaling Technology, Danvers, MA), and β-actin (1:2500, Cat# ab8226, Abcam, Cambridge, MA). The secondary antibodies used were HRP conjugated anti-rabbit, and anti-mouse antibodies (Jackson ImmunoResearch, West Grove, PA). Protein bands were quantified using ImageJ software (NIH, Bethesda, MD). All data are shown as the trisomic to diploid ratio for Ts2 and 2N mice, or for DS patients and 2N control subjects.

### Proteomics

#### In-gel digestion

EVs samples (10 μg protein for each sample) isolated from the brains of three Ts2 mice and three littermate controls were run into a 4% SDS-PAGE gel until all proteins entered the gel and stacked together. After staining by EZ-Run™ Protein Gel Staining Solution (Fisher BioReagents™, Boston, PA) the gel bands were excised, reduced, alkylated, and digested in-gel with sequence grade modified trypsin (Promega, Madison, WI) as described [48]. Peptides were extracted by 50% acetonitrile in 5% formic acid, dried by vacuum centrifugation, and desalted using StageTips [43] packed with C18 beads.

#### Liquid chromatography-MS/MS analysis

A nanoflow HPLC instrument, EASY nLC 1000, was coupled directly to a Q Exactive™ HF Hybrid Quadrupole-Orbitrap Mass Spectrometer [47] (Thermo Fisher Scientific) with a nanoelectrospray ion source (Thermo Fisher Scientific). Capillary PicoFrit columns (New Objective, Woburn, MA) were packed in-house with PeproSil-Pur C18-AQ3 μm resin (Dr. Maisch GmbH). Peptides were separated by a C18-reversed phase column (15 cm long, 75 μm inner diameter) with a linear gradient of 3 to 40% acetonitrile in 0.1% formic acid at a flow rate of 250 nL/min over 120 min. The Q Exactive™ HF was operated in top 15 data-dependent mode with dynamic exclusion duration of 30 s. Survey scans were acquired at a resolution of 120,000 at m/z 200 and HCD spectra at a resolution of 15,000 at m/z 200. Normalized collision energy was 27 eV.

#### MS data processing and analysis

The data analysis was performed with MaxQuant [13] software (Version 1.2.7.0, Max Planck Institute of Biochemistry) supported by Andromeda [14] to search a UniProt Mouse fasta database with 50,316 protein entries. Mass tolerance was set to 7 ppm for peptide masses and 20 ppm for HCD fragment ion masses with carbamidomethylation as a fixed modification and protein N-terminal acetylation and methionine oxidation as variable modifications. Up to two missed cleavages were allowed while requiring strict trypsin specificity and minimum sequence length of seven. Peptides and proteins were identified with a false discovery rate (FDR) of 1%. GO term enrichment analysis was done using Panther (http://geneontology.org/).

### Quantification of mRNA levels

qPCR was performed on microdissected mouse hippocampi with RNA purified using the miRNAeasy mini kit (Qiagen, Germantown, MD). Taqman qPCR primers for CD63 (Mm01966817_g1) and rab35 (Mm01204416_ml) (Life Technologies) were utilized with samples assayed on a real-time qPCR cycler (7900HT, Life Technologies) in 96-well optical plates with coverfilm as described previously [2–4, 18, 29]. The ddCT method was employed to determine relative gene level differences between Ts2 and 2N mice [1, 3, 4, 29]. Succinate Dehydrogenase Complex Flavoprotein Subunit A (*Sdha*, Mm01352360_m1) qPCR products were used as a control housekeeping gene. Negative controls consisted of the reaction mixture without input RNA and reaction mixture without Superscript III enzyme. PCR product synthesis was modeled as a function of mouse genotype, using mixed effects models to account for the correlation between repeated assays on the same mouse [37].

### Cell culture and interference RNA (siRNA) depletion

Forearm skin fibroblasts from DS patients (Cat#AG076985, Coriell Institute for medical research) and age-matched 2N control subjects (#AG06922, Coriell Institute for medical research) were grown in Dulbecco’s modified Eagle’s medium (DMEM, Thermo Fisher Scientific) supplemented with 10% fetal bovine serum (FBS, Thermo Fisher Scientific), 100 units/ml of penicillin and 100 μg/ml streptomycin (Thermo Fisher Scientific), 2 mM GlutaMAX (Thermo Fisher Scientific) at 5% CO_2_ at 37 °C in humidified air. Experiments were performed on DS and 2N cells with passage numbers from P8 to P14. Cells were seeded in P100 dishes with 12 mm coverslips at 300,000 (2N) or 350,000 (DS) cells per dish. The difference in the number of cells is because of a difference in division time. Transfection of siRNA was performed by combining 8 μl of siPORT Amine agent (Thermo Fisher Scientific) in 250 μl Opti-MEM (Thermo Fisher Scientific) with 250 μl Opti-MEM containing either CD63 siRNA (Cat# hs.Ri.CD63.13.2, Integrated DNA Technologies, Coralville, IA) or negative control siRNA (Integrated DNA Technologies). Cells were incubated with transfection complexes for 4 h and the medium was replaced with medium containing EVs-depleted FBS as described below.

### EVs isolation from fibroblast-conditioned media and quantification of exosome secreted levels

EVs were isolated from conditioned media of DS and diploid controls fibroblasts. Cell culture media were replaced with DMEM supplemented with 10% FBS, which was depleted of EVs by ultracentrifugation at 100,000 g for 70 min, 100 units/ml of penicillin and 100 μg/ml streptomycin (Thermo Fisher Scientific), and 2 mM GlutaMAX (Thermo Fisher Scientific). The conditioned media were collected every 24 h and fresh medium was added to the cells over 3 days. EVs were isolated from the conditioned media as previously described [50]. Briefly, conditioned media were centrifuged at 300 g for 10 min. The supernatant was sequentially centrifuged at 2000 g for 10 min, at 10,000 g for 30 min, and finally at 100,000 g to pellet the EVs. The EVs were resuspended in cold PBS and then centrifuged at 100,000 g. The washed EVs pellet was resuspended in PBS and lysated in 2X radioimmune precipitation assay (RIPA) buffer. Equal volumes of EVs lysates were loaded on the electrophoresis gel. Exosome levels were quantified by intensity of the bands obtained by Western-blot analysis of the exosomal markers CD63, Alix, and TSG101 using the ImageJ software (NIH).

### Morphometric analyses of endosomes

DS and 2N control fibroblasts grown on glass coverslips were fixed with cold 2% PFA in PBS for 20 min, subsequently blocked with 10% FBS for 1 h at room temperature, and incubated overnight with the anti-EEA1 antibody (1:200, Cat# 07–1820, EMD Millipore) at 4 °C. Cells were incubated with the secondary antibody Alexa 568 (1:500, Cat# A11036, Thermo Fisher Scientific) for 1 h at room temperature. Confocal microscopy was carried out using a LSM 510 microscope (Carl Zeiss). For the quantification of EEA1 staining, digital images were taken at ×63 magnification with comparable background intensities among conditions. The number of EEA1-positive endosomes and the endosomal area per cell were analyzed for each fibroblast in captured images taken in a single plane of focus by the ImageJ morphometry software (NIH) by a genotype- and condition-blinded observer. Individual fibroblasts and EEA1-positive endosomes were outlined and area measured. For each condition, a total of 20 fibroblasts per experiment were assessed at random from several fields.

### Statistical analyses

Data are presented as mean ± SEM. Unpaired, two-tailed, Student’s t-test statistical analysis were used to compare differences in EVs levels in DS brains and 2N controls, Ts2 brains and age-matched 2N controls, and to compare protein levels in Ts2 EVs, DS brain homogenates and DS fibroblast cell lysates to 2N controls. qPCR results were analyzed using one-way ANOVA and post-hoc analysis (Neumann-Keuls test; level of statistical significance was set at *p* < 0.05). Unpaired, two-tailed, Student’s t-test statistical analyses were used to determine in vitro differences in exosome secretion between untreated 2N with DS cells, and within the same line transfected with CD63 siRNA as compared to cells transfected with negative control siRNA. Endosomal changes in 2N and DS cells were assessed by one-way ANOVA followed by Tukey post-hoc multiple comparison test.

## Results

### Higher levels of EVs in the brain of DS patients and Ts2 mice compared to diploid controls

**Fig. 1:**
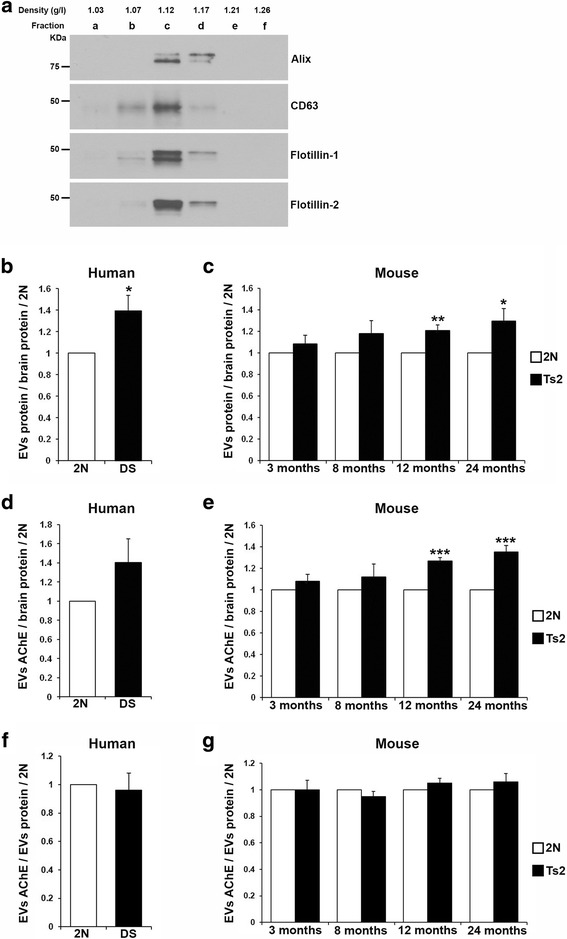
Higher levels of exosome-enriched EVs in the brains of DS patients and of Ts2 mice as compared to age-matched diploid controls. **a** Representative Western-blots of EVs isolated from human brain tissue and purified on a sucrose step gradient column. The sucrose gradient fractions b, c and d showed the presence of the exosomal proteins Alix and CD63, and the EVs proteins Flotillin-1 and Flotillin-2. **b** Quantification of total protein levels of EVs isolated from the brain extracellular space of DS patients, normalized to brain tissue protein levels, showed higher EVs levels compared to controls. **c** Higher EVs levels were also found in the brain extracellular space of 12- and 24-month-old Ts2 mice compared to 2N littermates. No significant differences were found in total EVs protein levels of 3- and 8-month-old Ts2 mice compared to controls. Similar results were obtained when AChE activity levels were measured in EVs isolated from the brain extracellular space of DS patients (**d**) and Ts2 mice (**e**) as compared to 2N controls when normalized to brain tissue protein content. AChE activity levels normalized to EVs protein content were not different between brains of DS patients (**f**) and Ts2 mice (**g**) compared to 2N controls. EVs levels are presented as trisomic to 2N ratio. Student t-test, *n* = 5 (DS and 2N human brains), *n* = 4 (3- and 24-month-old), *n* = 5 (8-month-old), and *n* = 7 (12-month-old) brains of Ts2 and 2N mice (*p < 0.05; **p < 0.01; ***p < 0.001)

Brain EVs were isolated as previously described [40, 41] from DS patients and age-matched normal controls (Table 1), and from the right hemibrain of 3-, 8-, 12- and 24-month-old Ts2 mice and 2N littermates. Separation of the EVs on a sucrose gradient resulted in 7 fractions, from a, the least dense, to g, the densest fraction, and Western-blot analysis showed that fractions with densities higher than 1.07 and lower than 1.17 (fractions b, c and d) were immunoreactive to Flotillin-1 and Flotillin-2, lipid raft proteins found in EVs, and established exosomal markers (Fig. 1a). Quantification of the exosome-enriched EVs fractions b, c, and d was performed by measuring the total protein content in the fractions normalized to total protein content in the brain tissue. In the samples of the frontal cortex of DS patients we found higher EVs levels compared to 2N controls (DS/2N ratio = 1.39, *p* = 0.022) (Fig. 1b). A similar increase in EVs levels was found in the brain extracellular space of the DS mouse model Ts2 at 12 (Ts2/2N ratio = 1.20, *p* = 0.0054) and 24 (Ts2/2N ratio = 1.29, *p* = 0.048) months of age compared to littermate controls, but not in younger, 3- (Ts2/2N ratio = 1.08, *p* = 0.31) and 8-month-old (Ts2/2N ratio = 1.18, *p* = 0.16) mice (Fig. 1c). We also measured the levels of exosome-enriched EVs by quantifying the activity of AChE, a protein that is specifically sorted into exosomes [27, 46]. The AChE activity measurements were normalized to total protein content in the brain tissue and the results supported the finding of DS-induced higher levels of exosomes with a trend in the brain extracellular space of DS patients (DS/2N ratio = 1.29, *p* = 0.14) (Fig. 1d), and conclusively in 12- (Ts2/2N ratio = 1.26, *p* = 0.00016) and 24-month-old (Ts2/2N ratio = 1.35, *p* = 0.00081) Ts2 mice (Fig. 1e). No significant differences in the levels of AChE activity were found in the brains of 3- (Ts2/2N = 1.08, *p* = 0.28) and 8-month-old (Ts2/2N = 1.12, *p* = 0.34) Ts2 mice compared to 2N littermates (Fig. 1e). Additionally, AChE activity was normalized to EVs protein content instead of total protein in the brain to estimate the AChE levels per EV. AChE activity levels per EV in the brains of DS patients were not different from 2N controls (DS/2N ratio = 0.96) (Fig. 1f), similar to the levels of AChE per EV in the brains of 3- (Ts2/2N ratio = 1.00), 8- (Ts2/2N ratio = 0.95), 12- (Ts2/2N ratio = 1.05) and 24-month-old (Ts2/2N ratio = 1.06) Ts2 mice compared to age-matched 2N controls (Fig. 1g). Altogether these results argue that higher number of exosome-enriched EVs loaded with similar levels of AChE are present in the brain extracellular space of DS patients and 12- and 24-month-old Ts2 mice compared to 2N controls.

### Higher content of CD63 and rab35, regulators of ILVs generation and release, in Ts2 brain EVs as compared to 2N

**Fig. 2:**
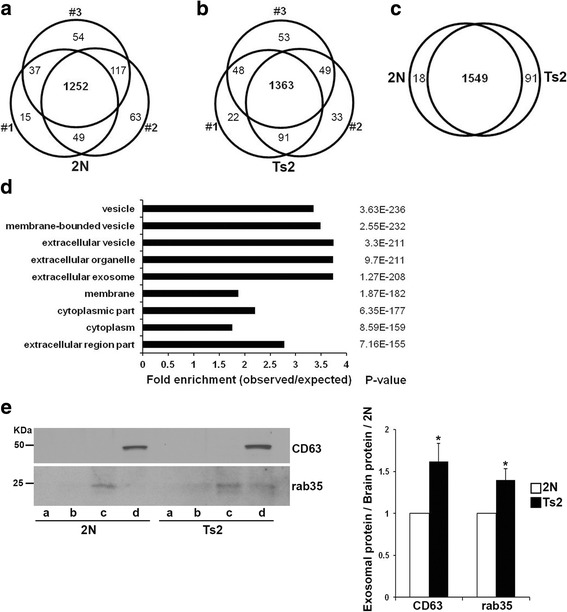
Proteomic analysis of exosome-enriched mouse brain EVs. A Venn diagram shows the overlap between biological replicates within each genotype for 2N (**a**) and Ts2 (**b**) EVs. **c** 1549 proteins were common to both genotypes. 91 and 18 proteins were unique to Ts2 and 2N samples, respectively. **d** GO analyses for components of the 1549 proteins common to both genotypes revealed enrichment of extracellular vesicles and exosomal proteins in the EVs preparations. *P*-values for each cellular category are shown on the right. **e** Representative Western-blots with anti-CD63 and anti-rab35 antibodies of the 2N and Ts2 sucrose gradient EVs fractions and corresponding quantification. Student t-test, *n* = 7 (*p < 0.05)

To identify candidate proteins whereby a change in expression could be mechanistically linked to increased levels of exosome-enriched EVs in DS, we performed label-free liquid chromatography-mass spectrometry (LC-MS)-based proteomics on EVs (pooled from the sucrose gradient fractions b, c, and d) isolated from the brains of 12-month-old Ts2 and 2N littermates. We identified 1587 and 1659 proteins in 2N and Ts2 EVs samples, respectively, with a large overlap in protein identification between biological replicates: 1252 common proteins in the samples from three 2N mice (Fig. 2a), and 1363 common proteins in the samples from three Ts2 mice (Fig. 2b). 1549 proteins were common to both genotypes (Fig. 2c). Gene ontology (GO) enrichment analysis of the common proteins identified by LC-MS showed that the most abundant components were proteins characteristic of vesicles, extracellular vesicles, extracellular organelles, and exosomes (Fig. 2d). Among the top 100 proteins that are often identified in exosomes (Exocarta, exosome database available online; http://www.exocarta.org/) 70 were identified in this study (Additional file 1: Table S1). Thus, using an unbiased approach, this analysis confirmed that vesicle-related proteins are highly enriched in brain EVs preparations, with specific enrichment of exosomal proteins.

The LC-MC analysis revealed a trend for higher levels of CD63 and rab35 in Ts2 EVs compared to 2N that was confirmed and found to be significantly different by Western-blot analysis (Fig. 2e).

### Higher protein expression levels of CD63 and rab35 in DS brains

**Fig. 3:**
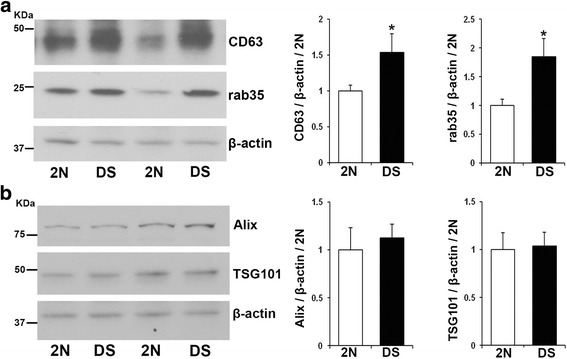
Enhanced expression of the proteins CD63 and rab35 in DS brains. **a** Representative Western-blots (corresponding to samples number 3, 2, 5 and 4 in Table 1) and quantification showing the overexpression of CD63 and rab35 in DS brains compared to 2N. **b** No differences in the levels of Alix or TSG101 were detected in homogenates of human DS brains compared to 2N controls. β-actin was blotted as an internal control for loading. Student t-test, *n* = 5 (*p < 0.05)

To determine whether the differences found in EVs protein levels reflect differences in protein expression levels within the cell leading to changes in exosome secretion, we analyzed the expression levels of CD63 and rab35 in human brain homogenates by Western-blot. Higher expression levels of CD63 (DS/2N ratio = 1.53; *p* = 0.030) and rab35 (DS/2N ratio = 1.85; *p* = 0.034) were observed in the frontal cortex of human DS patients as compared to 2N controls (Fig. 3a). In contrast, no significant differences were detected in the levels of the exosomal markers Alix (DS/2N ratio = 1.12; *p* = 0.66) and TSG101 (DS/2N ratio = 1.03; *p* = 0.88) between DS and 2N controls (Fig. 3b).

### Enhanced exosome secretion by DS fibroblasts

**Fig. 4:**
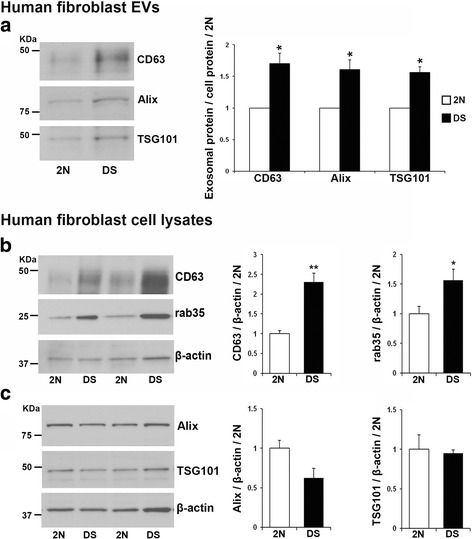
Exosome secretion is enhanced by DS fibroblasts as compared to 2N controls. **a** Representative Western-blots and corresponding quantification showing the higher levels of the exosomal markers CD63, Alix and TSG101 in EVs isolated from the conditioned media of DS fibroblasts compared to 2N controls. The intensity of the bands was normalized to cell protein. **b** Elevated expression levels of CD63 and rab35 and (**c**) no differences in the levels of Alix and TSG101 in lysates of DS fibroblasts compared to 2N controls, as shown by the representative Western-blots and corresponding quantification. β-actin was blotted as an internal control for loading. Student t-test, *n* = 5 independent experiments (*p < 0.05; **p < 0.01)

Given the endosomal abnormalities observed in cultured human forearm skin fibroblasts isolated from DS patients [8], similar to the brains of DS patients [10] and Ts2 mice [26], we hypothesized that exosome release into the media will be enhanced in order to alleviate the endosomal pathology. Western-blot analysis of EVs secreted into cultured media was performed with antibodies to CD63, rab35, Alix, and TSG101. Quantification of the bands and normalization to total cell proteins revealed that the levels of CD63 (DS/2N ratio = 1.70, *p* = 0.023), Alix (DS/2N ratio = 1.60, *p* = 0.030) and TSG101 (DS/2N ratio = 1.56, *p* = 0.022) were higher in DS EVs compared to 2N controls (Fig. 4a). The rab35 protein was not detected in fibroblasts EVs, potentially due to low content. We also studied the levels of these proteins in cell lysates and found that the expression levels of CD63 (DS/2N ratio = 2.30, *p* = 0.0016) and rab35 (DS/2N ratio = 1.56, *p* = 0.024) were higher in DS than in 2N fibroblasts (Fig. 4b), consistent with the observation in DS postmortem brain (Fig. 3a). However, the levels of Alix (DS/2N ratio = 0.62, *p* = 0.13) and TSG101 (DS/2N ratio = 0.95 *p* = 0.754) were not significantly different in the cell lysates obtained from DS as compared with 2N fibroblasts (Fig. 4c). Thus, the higher EVs levels of Alix and TSG101 in the media cultured by DS fibroblasts are due to higher levels of exosome secretion by DS fibroblasts as compared to 2N cells.

### Higher mRNA levels of CD63 but not rab35 in Ts2 brains

To determine whether differences in CD63 and rab35 protein levels were due to differences in mRNA levels, qPCR analyses were performed on RNA extracted from the hippocampus of 12-month-old Ts2 and 2N littermates. Upregulation of CD63 RNA expression was observed in Ts2 brains as compared with 2N controls (29% higher *p* = 0.0286; ddCt values expressed as mean ± SEM were 2.3558 ± 0.1966 in 2N and 3.0382 ± 0.1954 in DS; *n* = 5 per genotype). No significant difference was found for rab35 transcript levels.

### A role for CD63 in exosome secretion as a mechanism to modulate endosomal pathology

**Fig. 5:**
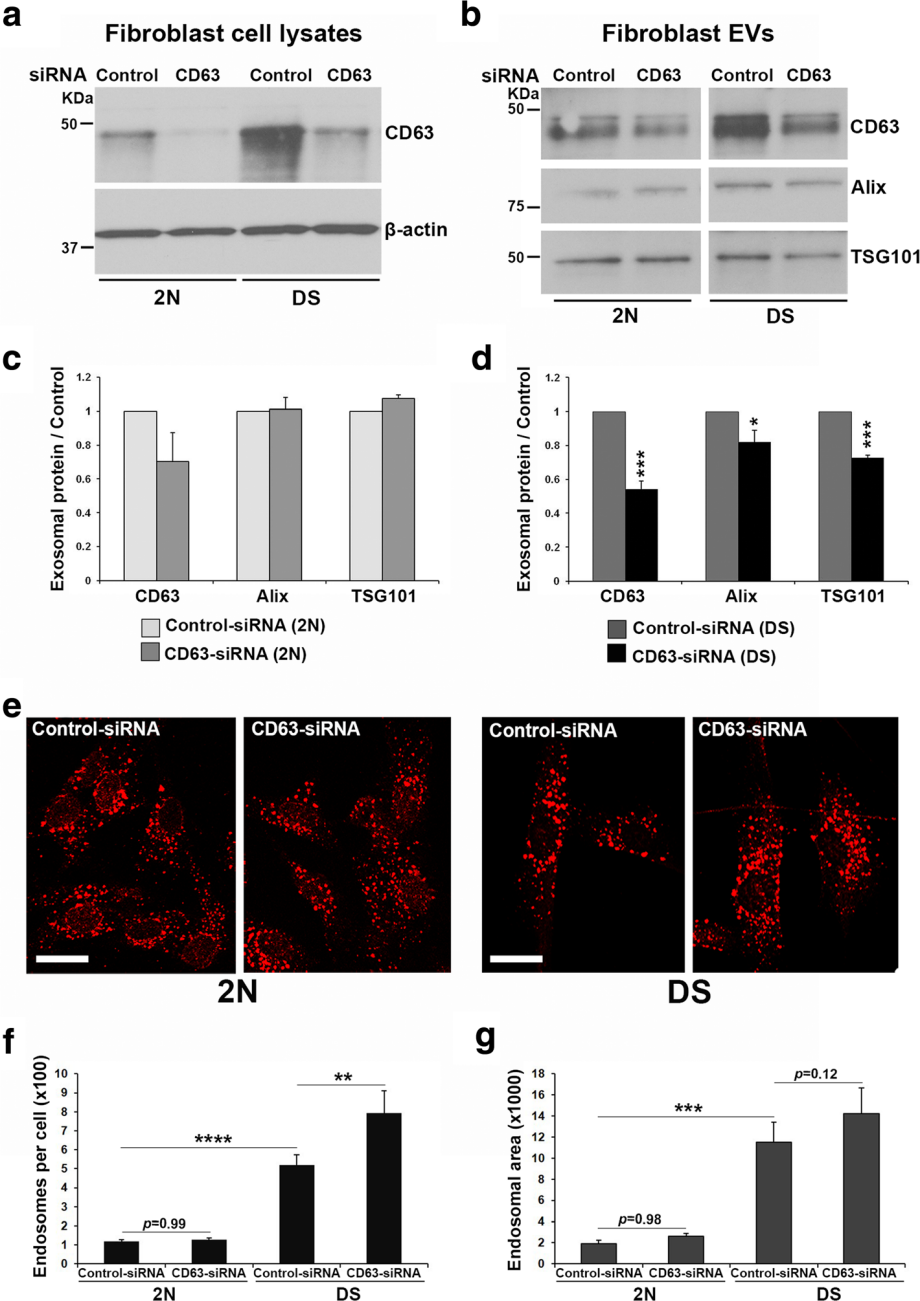
Effect of CD63 knockdown on exosome secretion and endosomal pathology in DS cells. 2N and DS fibroblasts were transfected with either CD63 or negative control siRNAs. **a** CD63 knockdown was confirmed by Western-blot analysis of cell lysates. **b** Over 3 days, exosomes were collected from the cell culture media and quantified by Western-blot analysis for the exosomal markers CD63, TSG101, and Alix. **c** No significant changes were observed in exosome release by 2N cells following CD63 silencing compared to controls. **d** DS fibroblasts in which CD63 was silenced showed decreased release of exosomes as seen by lower levels of exosomal TSG101 and Alix as compared to control DS cells. Student t-test, *n* = 4 independent experiments (*p < 0.05; ***p < 0.001). **e** Early endosomes were immunolabeled with an anti-EEA1 antibody of transfected 2N and DS cells (calibration bar = 20 μm). **f** No significant changes in the endosomal number were detected in CD63-reduced 2N fibroblasts compared to control 2N cells, while a significant increase in number of endosomes was observed in DS cells following CD63 knockdown. **g** No significant differences were found in the area occupied by endosomes in 2N and DS cells after knocking down CD63, however DS fibroblasts showed a trend for an increase. Note that the number and area occupied by endosomes in DS fibroblasts is significantly higher than in 2N under basal (control-siRNA) conditions (**f, g**). Area is expressed in pixels per cell. One-way ANOVA followed by Tukey post-hoc multiple comparison test, *n* = 4 independent experiments (**p < 0.01; ***p < 0.001; ****p< 0.0001)

We induced a CD63 loss of function in fibroblasts to investigate the role played by CD63 in the regulation of exosome secretion. Our small interfering RNA (siRNA) strategy targeting CD63 transcripts successfully knocked down CD63 levels in 2N and DS fibroblasts (Fig. 5a). The reduction in CD63 expression was 77.68 ± 5.1% and 73.03 ± 3.9% for 2N and DS cells, respectively. The exosomes secreted into the medium over the course of three days after the transfection were quantified by Western-blot analysis (Fig. 5b). No significant changes were found in exosome secretion by 2N cells following CD63 knockdown as evidenced by similar levels of Alix (CD63/control siRNA ratio = 1.01, *p* = 0.37) and TSG101 (CD63/control siRNA ratio = 1.07, *p* = 0.25) (Fig. 5c). The levels of CD63 in 2N EVs showed a trend to decrease (CD63/control siRNA ratio = 0.70, *p* = 0.08), which was expected due to CD63 knockdown. In contrast, reducing CD63 expression led to lower levels of exosomes secreted into the cell culture media by DS fibroblasts as seen by reduced levels of exosomal Alix (CD63/control siRNA ratio = 0.82, *p* = 0.02), TSG101 (CD63/control siRNA ratio = 0.73, *p* = 3.7 × 10^−7^) and, as expected, CD63 (CD63/control siRNA ratio = 0.56, *p* = 4.8 × 10^−6^) (Fig. 5d). When the expression levels of Alix and TSG101 were normalized to EVs protein content, no changes were detected in the levels of Alix (CD63/control siRNA ratio = 1.04, *p* = 0.69) and TSG101 (CD63/control siRNA ratio = 0.95, *p* = 0.76) in EVs from DS cells in which CD63 was reduced compared to controls, indicating that the loading levels of Alix and TSG101 per EV are similar after CD63 knockdown in DS cells. Therefore, the lower intensity of the bands for Alix and TSG101 shown in Fig. 5b is the result of lower number of EVs and rules out the possibility that CD63 knockdown is responsible for a change in EVs composition.

To determine whether changes in exosome secretion affect endosomal compartments, endosomal morphology was studied in 2N and DS fibroblasts using early endosome antigen 1 (EEA1) immunocytochemistry (Fig. 5e). As previously demonstrated [8], the number (Fig. 5f) and area (Fig. 5g) of endosomes were significantly higher in DS cells compared to 2N. While CD63 knockdown did not affect endosomes in 2N cells (Fig. 5f-g), the number of endosomes was higher (Fig. 5f) and a trend to increased endosomal area (Fig. 5g) was observed in DS fibroblasts in which CD63 expression was reduced compared to DS cells transfected with negative control siRNA.

## Discussion

Different types of EVs have distinct intracellular origin, including plasma membrane-derived microvesicles, apoptotic vesicles, and MVBs-derived exosomes (reviewed in [28, 30, 51]). We found higher levels of EVs in the brain extracellular space of DS patients and Ts2 mice compared to controls. Proteomic analysis confirmed that exosomal proteins were highly enriched in the brain EVs preparations, and identified higher levels of the exosomal marker CD63 and the exosome-related protein rab35 in brain-derived Ts2 EVs compared to 2N. These LC-MS data suggest that the higher levels of EVs found in the brain extracellular space of DS patients and Ts2 are due to higher levels of exosomes. The brains of DS patients at advanced ages likely have significant AD neuropathology, not present in age-matched control brains, raising the possibility that enhanced exosome secretion is due to AD neuropathology. However, we have previously reported that Tg2576 mice, overexpressing APP, do not secrete more exosomes than their littermate controls at an age when amyloid pathology has fully developed [40], suggesting that AD neuropathology is not causing higher exosome secretion. Further, we found that DS fibroblasts secrete more exosomes into the cell culture media than 2N cells. This suggests that the higher exosome levels found in vivo is a consequence of enhanced exosome secretion rather than altered exosome stability or less exosome clearance in the brain extracellular space.

**Fig. 6:**
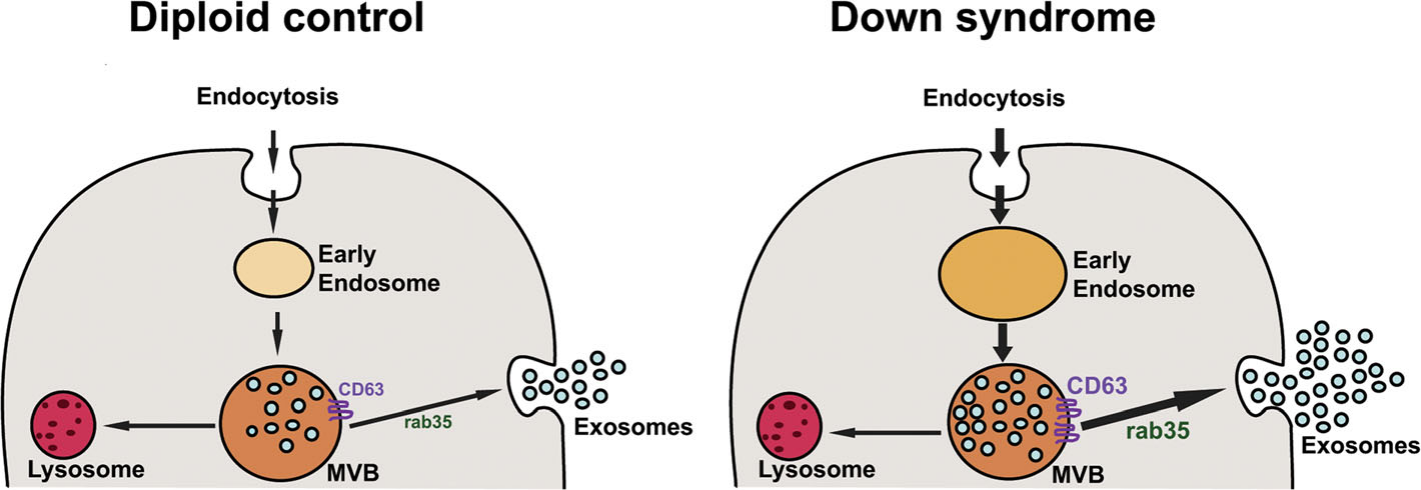
Schematic representation of the endosomal and exosomal pathways in diploid and DS neurons. Endocytosed material in the cell is transported by early endosomes and late endosomes/multivesicular bodies (MVBs) for either degradation in lysosomes or exosome secretion. The invagination of the MVBs membrane results in the formation of intraluminal vesicles (ILVs), which are released as exosomes into the extracellular space upon fusion of MVBs with the plasma membrane. Our findings show that in DS neurons with endosomal enlargement there is an enhanced exosome release regulated by the tetraspanin CD63

Once early endosomal cargoes are delivered to late endosomes there are two possible fates, either lysosome degradation or exosome release. The endosomal function under conditions of increased early endosomal drive in DS [8] would require a corresponding increase in either or both of these pathways. Since we measured a statistically enhanced exosome secretion in the brain of 12-month-old and older Ts2 mice and the endosome enlargement phenotype is observed in neurons of 4-month-old mice [26], we hypothesize that enhanced exosome secretion constitutes a delayed cellular response designed to lower the size and number of endosomal compartments in DS by shedding more endosomal content into the brain extracellular space (Fig. 6). A similar mechanism of exosome release was suggested for the cell to partially overcome the accumulation of free cholesterol within late endosomes/lysosomes in the Niemann-Pick Type C disease [49].

The expression levels of the ESCRT proteins Alix and TSG101 did not differ in the brains of human DS patients and in DS fibroblasts as compared with 2N controls. These data suggest that the ESCRT machinery is not the trigger behind enhanced exosome secretion. We found that CD63 is overexpressed in DS brains and in DS fibroblasts compared to 2N controls. Similarly, a recent study reported higher protein levels of another member of the tetraspanin family, tetraspanin-6, in the brains of AD patients, and in vitro experiments related tetraspanin-6 overexpression to the generation of more exosomes [19]. Moreover, we report that CD63 transcription is enhanced in the brains of Ts2 mice. In contrast, higher levels of protein expression, but not mRNA was found for rab35, a regulator of exosome secretion [24]. We suggest that higher rab35 protein levels may be downstream of the induction of CD63, in response to ILVs accumulation by releasing excess MVBs load into the extracellular space. Upregulation of CD63 gene rules out the possibility that higher CD63 protein levels in DS is due to its accumulation as a consequence of endosomal pathology. We then investigated whether enhanced exosome secretion by CD63 has a role in alleviation of endosomal pathology. DS fibroblasts secreted more exosomes into the cultured media compared to 2N cells and CD63 knockdown reduced exosome secretion by the DS cells. In support of an inter-relationship between exosomal release and endosomal pathology, CD63 knockdown also caused changes in endosomes of DS fibroblasts, characteristic of the endosomal abnormalities reported in DS patients [10], DS fibroblasts [8], and in DS mouse models [9, 26]. These data argue that partially blocking exosome release in cells with endosomal pathology aggravates the intracellular accumulation of endosomal membranes. In contrast, in normal cells without endosomal pathology, silencing CD63 did not affect exosome release or endosome accumulation, implying that exosome release can be regulated in 2N cells even under changes in expression of proteins involved in exosome generation, while DS cells lose this capability. Therefore, these data suggest that CD63 is involved in the formation of more ILVs in MVBs when the system is compromised, similar to the finding that CD63 overexpression is associated with higher exosome release in fibroblasts from patients with systemic sclerosis [38]. It cannot be ruled out that in the diseased brain, this protective mechanism of the exosome secretory pathway to relieve DS neurons of accumulated endosomal contents might be outweighed by the propagation of toxic material by exosomes throughout the brain. The ability of exosomes to promote the spread of the disease has emerged as a general mechanism of propagation of neurodegenerative disorders [23, 56] and it was recently reported that neuronal exosomes obtained from blood of DS patients have elevated levels of amyloid-β peptides and phosphorylated-tau [21]. Further, reducing exosome secretion has been suggested as a potential therapeutic intervention for AD [6, 16]. However, blocking exosome production resulted in exacerbated behavioral and pathological defects in a transgenic mouse overexpressing a mutant form of the toxic protein TDP-43 [25], negating inhibition of exosome release in neurodegenerative disorders as a therapeutic approach.

## Conclusions

In summary, the data presented here show a role for exosomes in the regulation of endosomal function in DS, implicate CD63 in driving exosome release in the DS brain, and suggest that this is a protective mechanism to alleviate the endosomal pathology. Since the naturally occurring enhancement of exosome release in the brain of DS patients is not sufficient to alleviate the endosomal pathology, which is already observed in fetuses and worsens with age, therapeutic approaches that enhance exosome secretion even further may be beneficial. Indeed, apart from pathological proteins, exosomes naturally transport cargoes with therapeutic properties [31, 33, 34, 45] and can be engineered to target neurons while carrying specific molecules for therapeutics [5]. Increasing our knowledge of brain exosome secretion and its potential therapeutic effects is necessary to provide new insights into the mechanisms of disease and may help to develop novel therapeutic strategies for neurodegenerative disorders with accumulation of toxic material in endosomes, like DS and AD.

## Additional file

Additional file 1: Table S1. Proteins identified by LC-MS analysis of brain EVs that are part of the top 100 proteins frequently identified in exosomes (http://exocarta.org/exosome_markers_new). (DOC 149 kb)

## Abbreviations

2N: normal disomic
AChE: Acetylcholine esterase
AD: Alzheimer’s disease
BA9: Brodmann area 9
DMEM: Dulbecco’s modified Eagle’s medium
DS: Down Syndrome
ESCRT: Endosomal sorting complexes required for transport
EVs: Extracellular vesicles
FBS: Fetal bovine serum
HA: Hibernate A
ILVs: Intraluminal vesicles
MVBs: Multivesicular bodies
