# Supplementary material for: Enhanced exosome secretion in Down syndrome brain - a protective mechanism to alleviate neuronal endosomal abnormalities
Source: Acta Neuropathol Commun. 2017 Aug 29;5:65. doi: 10.1186/s40478-017-0466-0 (PMC5576289; doi:10.1186/s40478-017-0466-0)
Supplement: Additional file 1: Table S1. — Proteins identified by LC-MS analysis of brain EVs that are part of the top 100 proteins frequently identified in exosomes (http://exocarta.org/exosome_markers_new). (DOC 149 kb) [file 40478_2017_466_MOESM1_ESM.doc]

| **#** | **Gene Symbol** | **Number of times identified in the literature** | **Identified in this study** |
| --- | --- | --- | --- |
| **1** | [CD9](http://exocarta.org/query_results?query_name=CD9&prot_name_s=&prot_name_c=) | 98 | Yes |
| **2** | [HSPA8](http://exocarta.org/query_results?query_name=HSPA8&prot_name_s=&prot_name_c=) | 97 | Yes |
| **3** | [PDCD6IP](http://exocarta.org/query_results?query_name=PDCD6IP&prot_name_s=&prot_name_c=) | 96 |  |
| **4** | [GAPDH](http://exocarta.org/query_results?query_name=GAPDH&prot_name_s=&prot_name_c=) | 95 | Yes |
| **5** | [ACTB](http://exocarta.org/query_results?query_name=ACTB&prot_name_s=&prot_name_c=) | 93 | Yes |
| **6** | [ANXA2](http://exocarta.org/query_results?query_name=ANXA2&prot_name_s=&prot_name_c=) | 83 | Yes |
| **7** | [CD63](http://exocarta.org/query_results?query_name=CD63&prot_name_s=&prot_name_c=) | 82 | Yes |
| **8** | [SDCBP](http://exocarta.org/query_results?query_name=SDCBP&prot_name_s=&prot_name_c=) | 78 |  |
| **9** | [ENO1](http://exocarta.org/query_results?query_name=ENO1&prot_name_s=&prot_name_c=) | 78 | Yes |
| **10** | [HSP90AA1](http://exocarta.org/query_results?query_name=HSP90AA1&prot_name_s=&prot_name_c=) | 77 | Yes |
| **11** | [TSG101](http://exocarta.org/query_results?query_name=TSG101&prot_name_s=&prot_name_c=) | 76 |  |
| **12** | [PKM](http://exocarta.org/query_results?query_name=PKM&prot_name_s=&prot_name_c=) | 72 |  |
| **13** | [LDHA](http://exocarta.org/query_results?query_name=LDHA&prot_name_s=&prot_name_c=) | 72 | Yes |
| **14** | [EEF1A1](http://exocarta.org/query_results?query_name=EEF1A1&prot_name_s=&prot_name_c=) | 71 |  |
| **15** | [YWHAZ](http://exocarta.org/query_results?query_name=YWHAZ&prot_name_s=&prot_name_c=) | 69 |  |
| **16** | [PGK1](http://exocarta.org/query_results?query_name=PGK1&prot_name_s=&prot_name_c=) | 69 |  |
| **17** | [EEF2](http://exocarta.org/query_results?query_name=EEF2&prot_name_s=&prot_name_c=) | 69 |  |
| **18** | [ALDOA](http://exocarta.org/query_results?query_name=ALDOA&prot_name_s=&prot_name_c=) | 69 |  |
| **19** | [HSP90AB1](http://exocarta.org/query_results?query_name=HSP90AB1&prot_name_s=&prot_name_c=) | 67 | Yes |
| **20** | [ANXA5](http://exocarta.org/query_results?query_name=ANXA5&prot_name_s=&prot_name_c=) | 67 | Yes |
| **21** | [FASN](http://exocarta.org/query_results?query_name=FASN&prot_name_s=&prot_name_c=) | 66 | Yes |
| **22** | [YWHAE](http://exocarta.org/query_results?query_name=YWHAE&prot_name_s=&prot_name_c=) | 65 | Yes |
| **23** | [CLTC](http://exocarta.org/query_results?query_name=CLTC&prot_name_s=&prot_name_c=) | 64 | Yes |
| **24** | [CD81](http://exocarta.org/query_results?query_name=CD81&prot_name_s=&prot_name_c=) | 64 | Yes |
| **25** | [ALB](http://exocarta.org/query_results?query_name=ALB&prot_name_s=&prot_name_c=) | 64 | Yes |
| **26** | [VCP](http://exocarta.org/query_results?query_name=VCP&prot_name_s=&prot_name_c=) | 62 | Yes |
| **27** | [TPI1](http://exocarta.org/query_results?query_name=TPI1&prot_name_s=&prot_name_c=) | 62 | Yes |
| **28** | [PPIA](http://exocarta.org/query_results?query_name=PPIA&prot_name_s=&prot_name_c=) | 62 | Yes |
| **29** | [MSN](http://exocarta.org/query_results?query_name=MSN&prot_name_s=&prot_name_c=) | 62 | Yes |
| **30** | [CFL1](http://exocarta.org/query_results?query_name=CFL1&prot_name_s=&prot_name_c=) | 62 | Yes |
| **31** | [PRDX1](http://exocarta.org/query_results?query_name=PRDX1&prot_name_s=&prot_name_c=) | 61 | Yes |
| **32** | [PFN1](http://exocarta.org/query_results?query_name=PFN1&prot_name_s=&prot_name_c=) | 61 |  |
| **33** | [RAP1B](http://exocarta.org/query_results?query_name=RAP1B&prot_name_s=&prot_name_c=) | 60 | Yes |
| **34** | [ITGB1](http://exocarta.org/query_results?query_name=ITGB1&prot_name_s=&prot_name_c=) | 60 | Yes |
| **35** | [HSPA5](http://exocarta.org/query_results?query_name=HSPA5&prot_name_s=&prot_name_c=) | 58 | Yes |
| **36** | [SLC3A2](http://exocarta.org/query_results?query_name=SLC3A2&prot_name_s=&prot_name_c=) | 57 | Yes |
| **37** | [HIST1H4A](http://exocarta.org/query_results?query_name=HIST1H4A&prot_name_s=&prot_name_c=) | 57 | Yes |
| **38** | [GNB2](http://exocarta.org/query_results?query_name=GNB2&prot_name_s=&prot_name_c=) | 57 | Yes |
| **39** | [ATP1A1](http://exocarta.org/query_results?query_name=ATP1A1&prot_name_s=&prot_name_c=) | 57 | Yes |
| **40** | [YWHAQ](http://exocarta.org/query_results?query_name=YWHAQ&prot_name_s=&prot_name_c=) | 56 | Yes |
| **41** | [FLOT1](http://exocarta.org/query_results?query_name=FLOT1&prot_name_s=&prot_name_c=) | 56 | Yes |
| **42** | [FLNA](http://exocarta.org/query_results?query_name=FLNA&prot_name_s=&prot_name_c=) | 56 |  |
| **43** | [CLIC1](http://exocarta.org/query_results?query_name=CLIC1&prot_name_s=&prot_name_c=) | 56 |  |
| **44** | [CDC42](http://exocarta.org/query_results?query_name=CDC42&prot_name_s=&prot_name_c=) | 56 | Yes |
| **45** | [CCT2](http://exocarta.org/query_results?query_name=CCT2&prot_name_s=&prot_name_c=) | 56 | Yes |
| **46** | [A2M](http://exocarta.org/query_results?query_name=A2M&prot_name_s=&prot_name_c=) | 55 |  |
| **47** | [YWHAG](http://exocarta.org/query_results?query_name=YWHAG&prot_name_s=&prot_name_c=) | 54 | Yes |
| **48** | [TUBA1B](http://exocarta.org/query_results?query_name=TUBA1B&prot_name_s=&prot_name_c=) | 53 |  |
| **49** | [RAC1](http://exocarta.org/query_results?query_name=RAC1&prot_name_s=&prot_name_c=) | 53 | Yes |
| **50** | [LGALS3BP](http://exocarta.org/query_results?query_name=LGALS3BP&prot_name_s=&prot_name_c=) | 53 |  |
| **51** | [HSPA1A](http://exocarta.org/query_results?query_name=HSPA1A&prot_name_s=&prot_name_c=) | 53 | Yes |
| **52** | [GNAI2](http://exocarta.org/query_results?query_name=GNAI2&prot_name_s=&prot_name_c=) | 53 | Yes |
| **53** | [ANXA1](http://exocarta.org/query_results?query_name=ANXA1&prot_name_s=&prot_name_c=) | 53 | Yes |
| **54** | [RHOA](http://exocarta.org/query_results?query_name=RHOA&prot_name_s=&prot_name_c=) | 52 | Yes |
| **55** | [MFGE8](http://exocarta.org/query_results?query_name=MFGE8&prot_name_s=&prot_name_c=) | 52 | Yes |
| **56** | [PRDX2](http://exocarta.org/query_results?query_name=PRDX2&prot_name_s=&prot_name_c=) | 51 | Yes |
| **57** | [GDI2](http://exocarta.org/query_results?query_name=GDI2&prot_name_s=&prot_name_c=) | 51 | Yes |
| **58** | [EHD4](http://exocarta.org/query_results?query_name=EHD4&prot_name_s=&prot_name_c=) | 51 | Yes |
| **59** | [ACTN4](http://exocarta.org/query_results?query_name=ACTN4&prot_name_s=&prot_name_c=) | 51 |  |
| **60** | [YWHAB](http://exocarta.org/query_results?query_name=YWHAB&prot_name_s=&prot_name_c=) | 50 | Yes |
| **61** | [RAB7A](http://exocarta.org/query_results?query_name=RAB7A&prot_name_s=&prot_name_c=) | 50 | Yes |
| **62** | [LDHB](http://exocarta.org/query_results?query_name=LDHB&prot_name_s=&prot_name_c=) | 50 | Yes |
| **63** | [GNAS](http://exocarta.org/query_results?query_name=GNAS&prot_name_s=&prot_name_c=) | 50 | Yes |
| **64** | [TFRC](http://exocarta.org/query_results?query_name=TFRC&prot_name_s=&prot_name_c=) | 49 | Yes |
| **65** | [RAB5C](http://exocarta.org/query_results?query_name=RAB5C&prot_name_s=&prot_name_c=) | 49 | Yes |
| **66** | [ARF1](http://exocarta.org/query_results?query_name=ARF1&prot_name_s=&prot_name_c=) | 49 | Yes |
| **67** | [ANXA6](http://exocarta.org/query_results?query_name=ANXA6&prot_name_s=&prot_name_c=) | 49 | Yes |
| **68** | [ANXA11](http://exocarta.org/query_results?query_name=ANXA11&prot_name_s=&prot_name_c=) | 49 | Yes |
| **69** | [ACTG1](http://exocarta.org/query_results?query_name=ACTG1&prot_name_s=&prot_name_c=) | 49 |  |
| **70** | [KPNB1](http://exocarta.org/query_results?query_name=KPNB1&prot_name_s=&prot_name_c=) | 48 |  |
| **71** | [EZR](http://exocarta.org/query_results?query_name=EZR&prot_name_s=&prot_name_c=) | 48 | Yes |
| **72** | [ANXA4](http://exocarta.org/query_results?query_name=ANXA4&prot_name_s=&prot_name_c=) | 48 | Yes |
| **73** | [ACLY](http://exocarta.org/query_results?query_name=ACLY&prot_name_s=&prot_name_c=) | 48 | Yes |
| **74** | [TUBA1C](http://exocarta.org/query_results?query_name=TUBA1C&prot_name_s=&prot_name_c=) | 47 |  |
| **75** | [RAB14](http://exocarta.org/query_results?query_name=RAB14&prot_name_s=&prot_name_c=) | 47 | Yes |
| **76** | [HIST2H4A](http://exocarta.org/query_results?query_name=HIST2H4A&prot_name_s=&prot_name_c=) | 47 |  |
| **77** | [GNB1](http://exocarta.org/query_results?query_name=GNB1&prot_name_s=&prot_name_c=) | 47 | Yes |
| **78** | [UBA1](http://exocarta.org/query_results?query_name=UBA1&prot_name_s=&prot_name_c=) | 46 | Yes |
| **79** | [THBS1](http://exocarta.org/query_results?query_name=THBS1&prot_name_s=&prot_name_c=) | 46 |  |
| **80** | [RAN](http://exocarta.org/query_results?query_name=RAN&prot_name_s=&prot_name_c=) | 46 |  |
| **81** | [RAB5A](http://exocarta.org/query_results?query_name=RAB5A&prot_name_s=&prot_name_c=) | 46 | Yes |
| **82** | [PTGFRN](http://exocarta.org/query_results?query_name=PTGFRN&prot_name_s=&prot_name_c=) | 46 | Yes |
| **83** | [CCT5](http://exocarta.org/query_results?query_name=CCT5&prot_name_s=&prot_name_c=) | 46 | Yes |
| **84** | [CCT3](http://exocarta.org/query_results?query_name=CCT3&prot_name_s=&prot_name_c=) | 46 | Yes |
| **85** | [BSG](http://exocarta.org/query_results?query_name=BSG&prot_name_s=&prot_name_c=) | 46 | Yes |
| **86** | [AHCY](http://exocarta.org/query_results?query_name=AHCY&prot_name_s=&prot_name_c=) | 46 | Yes |
| **87** | [RAB5B](http://exocarta.org/query_results?query_name=RAB5B&prot_name_s=&prot_name_c=) | 45 | Yes |
| **88** | [RAB1A](http://exocarta.org/query_results?query_name=RAB1A&prot_name_s=&prot_name_c=) | 45 | Yes |
| **89** | [LAMP2](http://exocarta.org/query_results?query_name=LAMP2&prot_name_s=&prot_name_c=) | 45 | Yes |
| **90** | [ITGA6](http://exocarta.org/query_results?query_name=ITGA6&prot_name_s=&prot_name_c=) | 45 |  |
| **91** | [HIST1H4B](http://exocarta.org/query_results?query_name=HIST1H4B&prot_name_s=&prot_name_c=) | 45 |  |
| **92** | [GSN](http://exocarta.org/query_results?query_name=GSN&prot_name_s=&prot_name_c=) | 45 |  |
| **93** | [FN1](http://exocarta.org/query_results?query_name=FN1&prot_name_s=&prot_name_c=) | 45 |  |
| **94** | [YWHAH](http://exocarta.org/query_results?query_name=YWHAH&prot_name_s=&prot_name_c=) | 44 | Yes |
| **95** | [TUBA1A](http://exocarta.org/query_results?query_name=TUBA1A&prot_name_s=&prot_name_c=) | 44 |  |
| **96** | [TKT](http://exocarta.org/query_results?query_name=TKT&prot_name_s=&prot_name_c=) | 44 |  |
| **97** | [TCP1](http://exocarta.org/query_results?query_name=TCP1&prot_name_s=&prot_name_c=) | 44 |  |
| **98** | [STOM](http://exocarta.org/query_results?query_name=STOM&prot_name_s=&prot_name_c=) | 44 | Yes |
| **99** | [SLC16A1](http://exocarta.org/query_results?query_name=SLC16A1&prot_name_s=&prot_name_c=) | 44 |  |
| **100** | [RAB8A](http://exocarta.org/query_results?query_name=RAB8A&prot_name_s=&prot_name_c=) | 44 | Yes |
